# Supplementary material for: Enhanced Electrochemical Performance of PEO-Based Composite Polymer Electrolyte with Single-Ion Conducting Polymer Grafted SiO2 Nanoparticles
Source: Polymers (Basel). 2023 Jan 11;15(2):394. doi: 10.3390/polym15020394 (PMC9866075; doi:10.3390/polym15020394)
Supplement: Supplementary file 1 [file polymers-15-00394-s001.zip › polymers-2079592-supplementary.pdf]

polymers MDPI

Supporting Information

Enhanced electrochemical performance of PEO-based composite polymer electrolyte with single-ion conducting polymer grafted SiO_2 nanoparticles

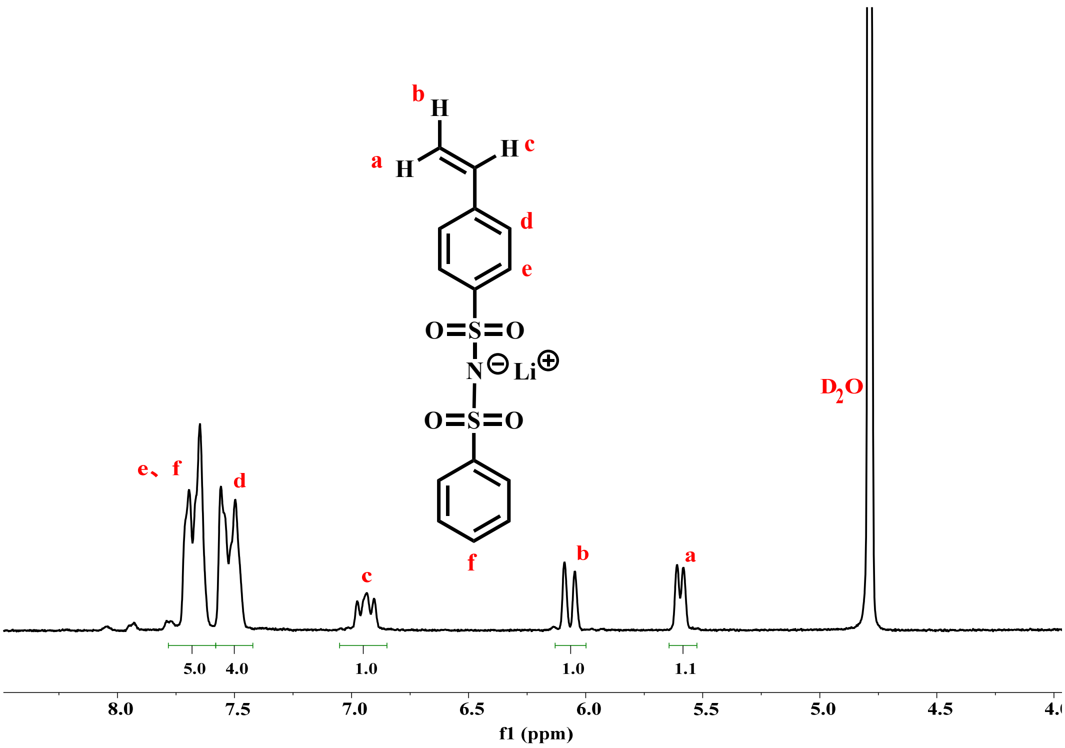

Figure S1. ^{1}H NMR spectrum of LiSSPSI in D_2O.

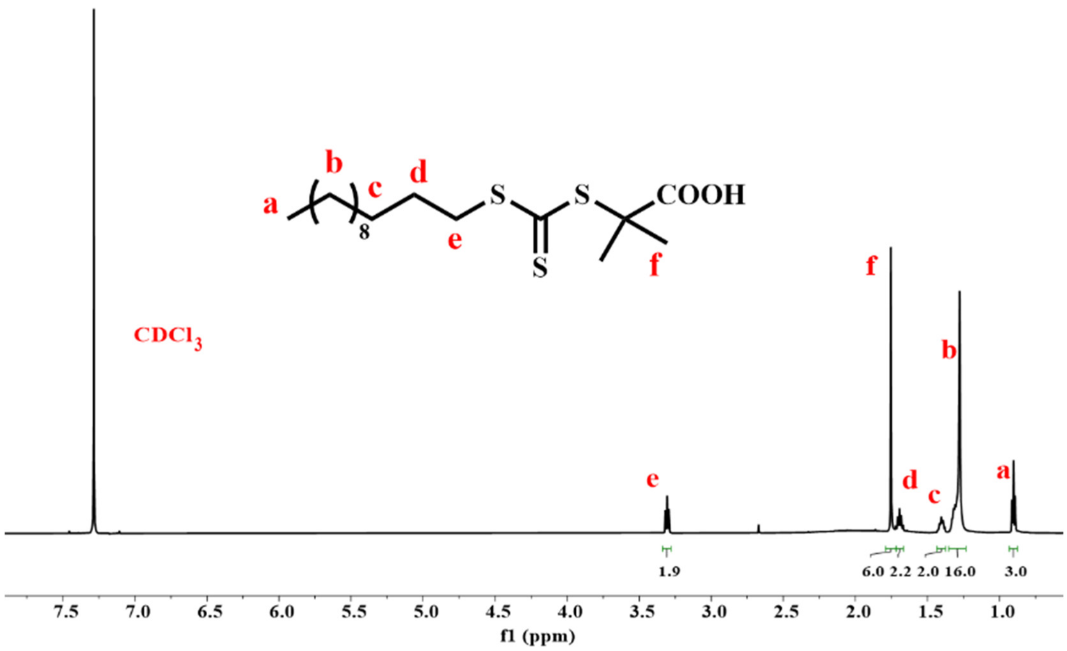

Figure S2. ^{1}H NMR spectrum of DTPA in $CDCl_3$.

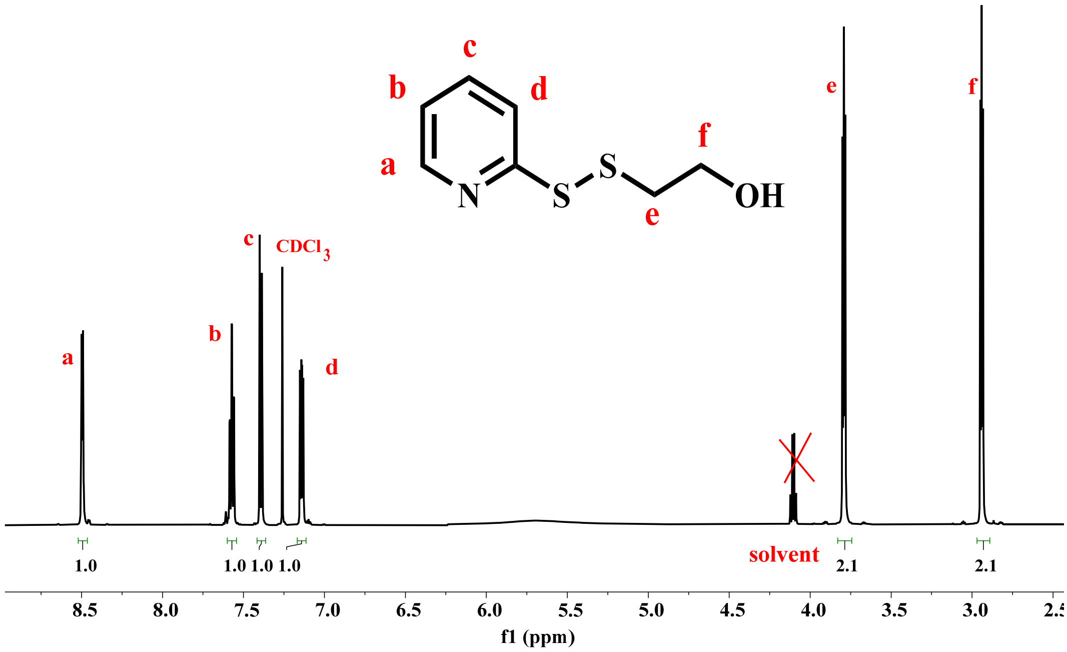

Figure S3. ^{1}H NMR spectrum of PDE in $CDCl_3$.

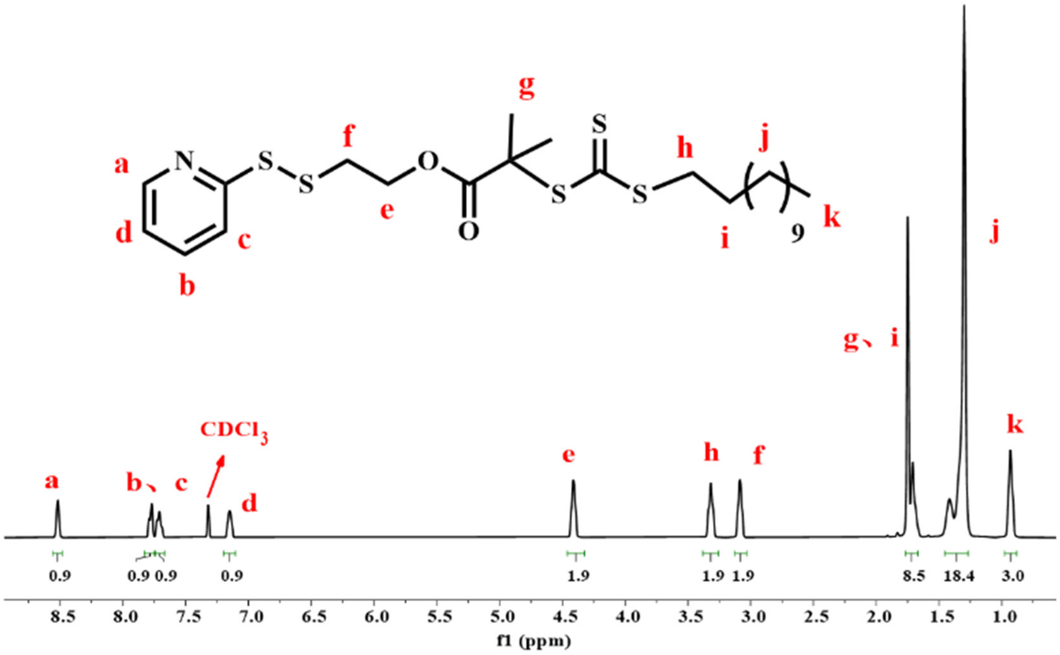

Figure S4. ^{1}H NMR spectrum of py-ss-DTPA in $CDCl_3$.

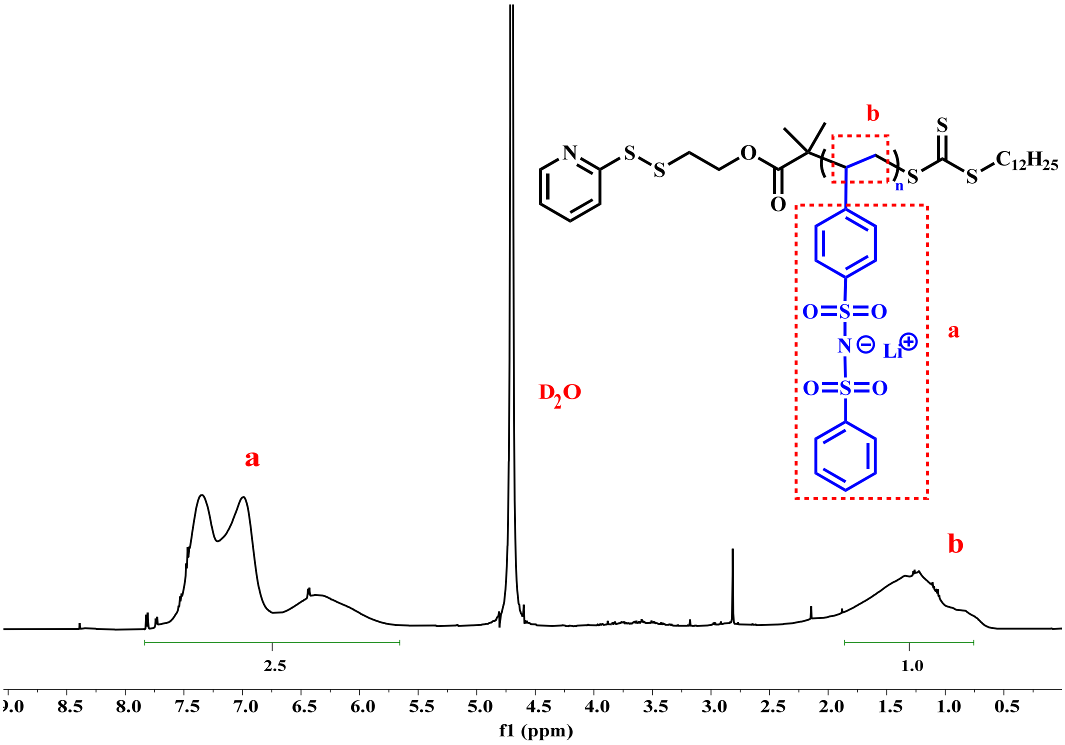

Figure S5. ^{1}H NMR spectrum of py-ss-PLiSSPSI in D_2O.

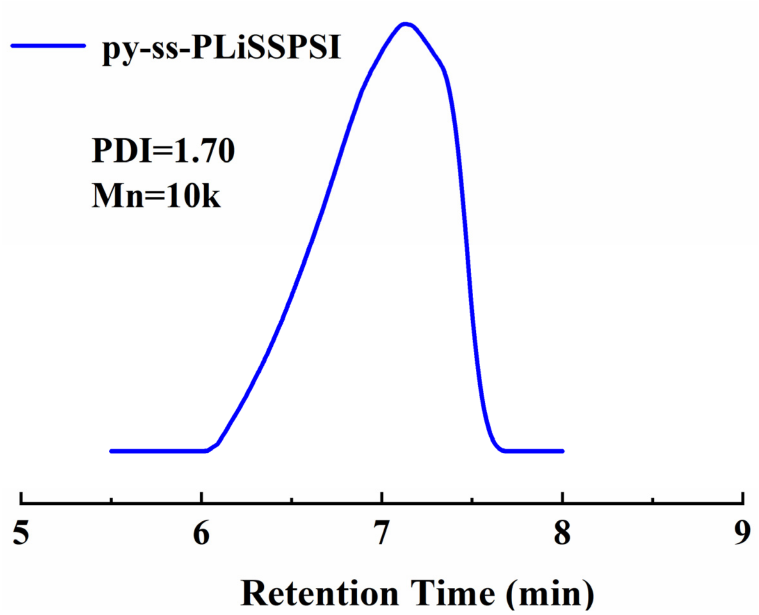

Figure S6. Overlays of SEC chromatograms (RI detection, in THF) for py-ss-PLiSSPSI.

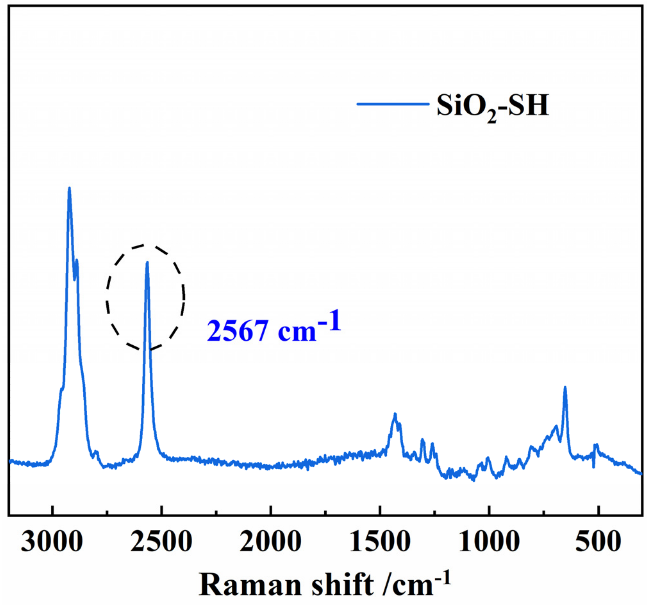

Figure S7. Raman spectrum of SiO_2-SH.

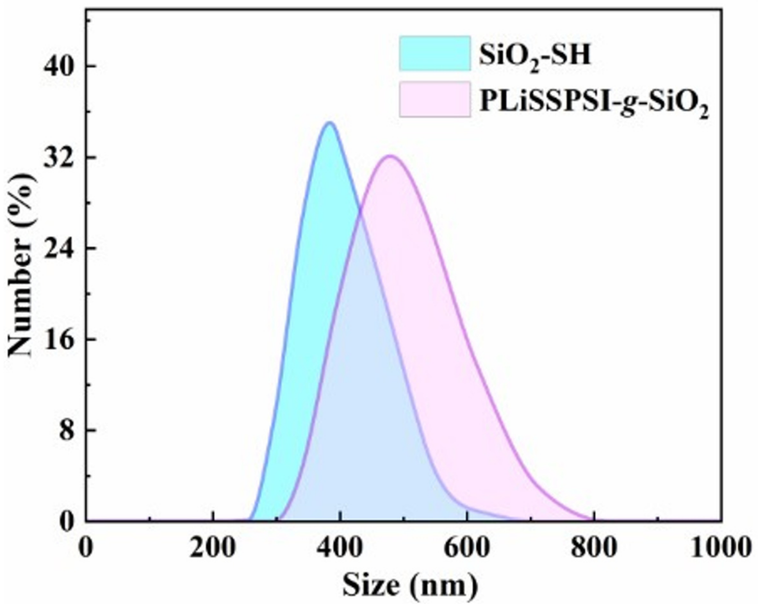

Figure S8. Particles size statistics of SiO_2-SH and PLiSSPSI-*g*-SiO_2.

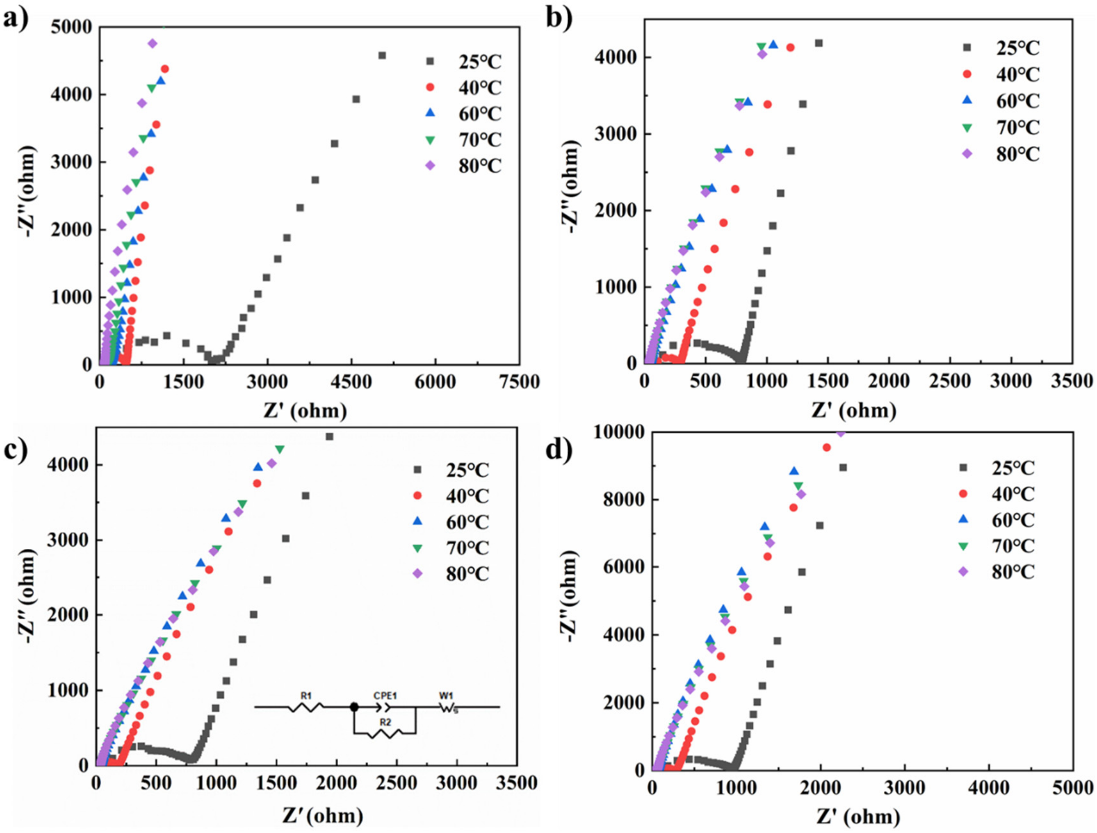

Figure S9. The impedance plots of SS|PLi-*g*-SiCSE-x|SS symmetric cells: a) PEO/LiTFSI, b) PLi-*g*-SiCSE-2, c) PLi-*g*-SiCSE-6, d) PLi-*g*-SiCSE-10.

Table S1. EIS fitting results of SS|PLi-*g*-SiCSE-6|SS.

	R1	R2	CPE-T	CPE-P	W1
Value	40.96	18.1	1.45E−9	0.91	168130
Error(%)	6.04	14.64	150.89	0.66	3.03

Table S2. The ionic conductivity (60 °C) of the prepared CSEs membranes containing PEO/LiTFSI and different amounts of PLiSSPSI-*g*-SiO_2 nanoparticle.

	PEO/LiTFSI	PLi-*g*-SiCSE-2	PLi-*g*-SiCSE-6	PLi-*g*-SiCSE-10
σ (mS·cm^{-1})	0.06	0.17	0.22	0.13
